# Three-Dimensional Trunk and Lower Limbs Characteristics during Gait in Patients with Huntington's Disease

**DOI:** 10.3389/fnins.2017.00566

**Published:** 2017-10-12

**Authors:** Elzbieta Mirek, Magdalena Filip, Wiesław Chwała, Krzysztof Banaszkiewicz, Monika Rudzinska-Bar, Jadwiga Szymura, Szymon Pasiut, Andrzej Szczudlik

**Affiliations:** ^1^Department of Clinical Rehabilitation and Laboratory of Pathology of the Musculoskeletal System, University School of Physical Education, Cracow, Poland; ^2^Department of Anthropomotorics, University School of Physical Education, Cracow, Poland; ^3^Department of Neurology and Neurorehabilitation, John Paul's II Hospital, Cracow, Poland; ^4^Department of Neurology, Medical University of Silesia, Katowice, Poland; ^5^Krakowska Akademia Neurologii, Cracow, Poland

**Keywords:** gait disorders, neurologic, Huntington's disease, kinematics parameters, biomechanical gait analysis, three dimensional motion analysis

## Abstract

**Objective:** A number of studies on gait disturbances have been conducted, however, no clear pattern of gait disorders was described. The aim of the study was to characterize the gait pattern in HD patients by conducting analysis of mean angular movement changes the lower limb joints and trunk (kinematics parameters).

**Methods:** The study group consisted of 30 patients with HD (17 women and 13 men). The reference data include the results of 30 healthy subjects (17 women and 13 men). Registration of gait with the Vicon 250 system was performed using passive markers attached to specific anthropometric points directly on the skin, based on the Golem biomechanical model (Oxford Metrics Ltd.). The research group and the control group were tested once.

**Results:** Statistically significant (*p* < 0.05) angular changes in gait cycle for HD patients were observed in: insufficient plantar flexion during Loading Response and Pre-swing phases; insufficient flexion of the knee joint during Initial Swing and Mid Swing phases; excessive flexion of the hip in Terminal Stance and Pre-swing phases and over-normative forward inclination of the trunk in all gait phases. It should be noted that the group of patients with HD obtained, for all the mean angular movement changes higher standard deviation.

**Conclusion:** A characteristic gait disorder common to all patients with HD occurring throughout the whole duration of the gait cycle is a pathological anterior tilt of the trunk. The results will significantly contribute to programming physiotherapy for people with HD, aimed at stabilizing the trunk in a position of extension during gait.

## Introduction

Huntington's disease (HD) is an autosomal dominant inherited disorder which is characterized by a triad of symptoms: movement disorders (chorea, dystonia, bradykinesia, parkinsonism, impaired gait, and balance), cognitive dysfunctions and behavioral disturbances. The first HD symptoms usually appear between the age of 35 and 44 and gradually progress leading to disability and death. The occurrence of HD falls between ~1.63 and 9.95 in 100,000 (Harper, 2002; Fahn et al., 2011).

Gait disturbances in the early stages of HD—the so-called “choreiform gait,” is a gait pattern mixed of unpredictable accelerations and decelerations in walking speed with superimposed twisting choreatic movements of the trunk, head, arms and legs. HD Gait is a mixture of chorea, myoclonus, ataxia, and probably most importantly, lack of rhythmic control and usually occurs on a widened base. In addition, the loss of postural reflexes is already well proven. In the later stages of HD, gait becomes stiff, cautious and slower (Bilney et al., 2005; Collett et al., 2014; Danoudis and Iansek, 2014).

A number of studies on gait disturbances have been conducted. Three-dimensional gait analysis showed shortening the of the gait cycle, increased duration of the dual-support phase and increased variability of the duration of the gait cycle and step as compared to the healthy controls (Koller and Trimble, 1985; Reynolds et al., 1999; Grimbergen et al., 2008; Rao et al., 2008). These results do not show the origin of gait and posture disturbances in the musculoskeletal system and thus, they play a minor role in planning the physiotherapy of HD patients.

Previous research analyze the selective evaluation of kinematic, spatiotemporal parameters or rhytmic control of gait in HD (Reynolds et al., 1999; Delval et al., 2006). However, in the present study, we analyzed the kinematic parameters of the lower limbs and the trunk, which represents a new approach to the assessment and discussion of gait disorders in HD.

The aim of the study was to characterize the gait pattern in HD patients by conducting analysis of mean angular movement changes of the lower limb joints, and trunk (kinematics parameters).

## Research materials and methods

The study group was selected from a cohort of patients who participated in the European Huntington Disease Network (EHDN) REGISTRY project at the Department of Neurology, University Hospital, Jagiellonian University in Krakow. The study was approved by The Bioethics Committee of the Jagiellonian University in Krakow (Approval No.: KBET/59B/2010).

Inclusion criteria were as follows: positive results of HD genetic testing, typical HD motor manifestation, optimal pharmacotherapy which was not changed for at least 1 month before inclusion, patient's written informed consent. The exclusion criteria were: any gait abnormality that made the subject unable to independently walk 20 m in a straight line, using an assistive device during gait, severe cognitive impairment (moderate and severe dementia), behavioral and/or psychotic disturbances preventing effective cooperation, orthopedic disorders, or traumatic injuries to the musculoskeletal system permanently disrupting the gait pattern and any other severe, chronic, or acute conditions that might significantly influence general health, e.g., cancer, myocardial infarction, chronic pulmonary disease.

Recruitment and all assessments were performed between July 2010 and December 2012. Participation in the study was proposed to all 32 patients who were registered in the REGISTRY project and met the inclusion criteria. Two patients did not give their written consent to participate in the project. Ultimately, the studied group consisted of 30 patients (17 women). The patients' optimal pharmacotherapy included neuroleptics, antidepressants and benzodiazepines.

All HD patients underwent neurological assessment according to the Unified Huntington's Disease Rating Scale (UHDRS) [Huntington Study Group (Kieburtz, K., primary author), 1996] and the Total Functional Capacity Scale (TFC) (Shoulson and Fahn, 1979), as well as 3-D gait registration and analysis using the Vicon 250 system. Neurological examination and qualification for the study was done by a neurologist. The characteristics of the group are presented in Table 1. HD patients were in early or moderately advanced stages of the disease. They were independent or required little help in everyday activities. There was significant patient-to-patient variability in terms of cognitive impairment.

**Table 1:** Neurological characteristics of the study group.

**Patients**	**Mean ±*SD***	**Range**
Number of CAG repetitions	46.6 ± 6.0	40–62
Disease duration [years]	7.0 ± 5.8	2–10
UHDRS cognitive [points]	123.9 ± 43.3	10–186
UHDRS motor [points]	40.8 ± 20.0	10–78
UHDRS functional assessment [points]	17.6 ± 4.6	7–25
UHDRS independence scale [points]	76.7 ± 13.2	50–100
TFC [points]	7.7 ± 3.1	3–13

*UHDRS, Unified Huntington's Disease Rating Scale*.

*TFC, Total Functional Capacity*.

*CAG, cytosine-adenine-guanine*.

Gait analysis data were compared to the results of a control group comprised of healthy volunteers from Krakow (they were not a family of HD patients). The data were collected at the Laboratory of Biokinetics Department of the Biomechanics Faculty of the University School of Physical Education in Krakow. The data include spatio-temporal and angular parameters of a normalized gait cycle in 30 healthy subjects (17 women). Inclusion criteria were as follows: not diagnosed with any neurological disorders, chronic diseases, or traumatic injury of the musculoskeletal system which could affect gait pattern. Since spatio-temporal parameters affect the angular parameters of gait, the control group was matched according to cadence and gait speed with the HD group. Exclusion criteria were as follows: gait speed ≥1.45 or ≤1.21 m/s, cadence ≥11.8 or ≤121.4 Hz. Characteristics of the study group and controls in terms of body build and gait parameters are shown in Table 2.

**Table 2:** Characteristics of the study and control group.

**Variable**	**Study group**	**Control group**
Age [years]	43.4 ± 13.8	48.7 ± 14.7
Body height [m]	1.67 ± 0.10	1.71 ± 0.10
Body mass [kg]	62.5 ± 17.6	73.6 ± 11.4
Walking speed [m/s]	1.07 ± 0.25	1.33 ± 0.12
Cadence [Hz]	107.6 ± 15.6	116.1 ± 5.3
Step length [m]	0.59 ± 0.11	0.69 ± 0.04

**Figure 1:**
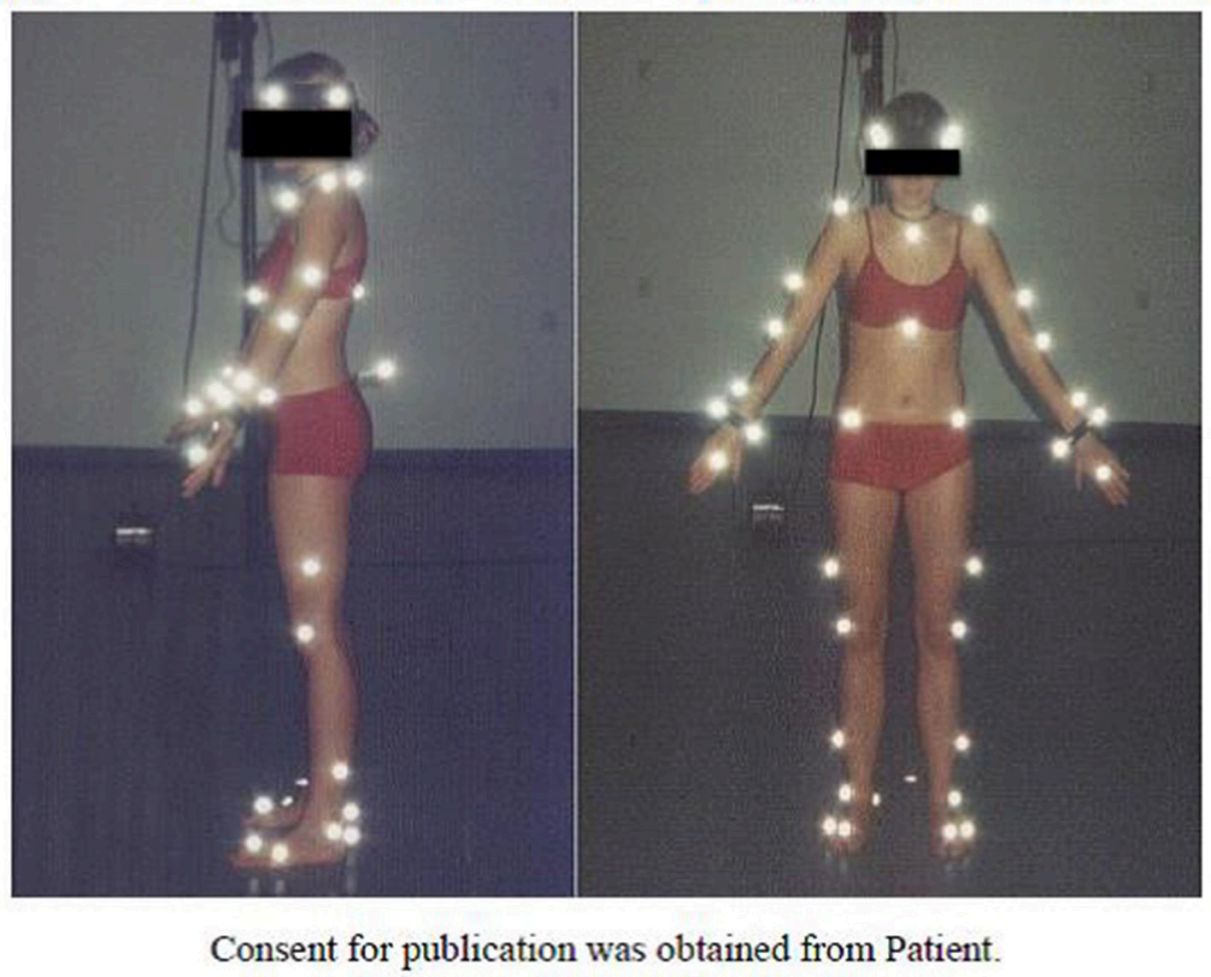
Passive markers on the examined person (side and front view).

Registration of gait was performed using passive markers attached to specific anthropometric points directly on the skin (Figure 1), based on the Golem biomechanical model (Oxford Metrics Ltd.). Gait registration was done with use of five cameras at a 120 Hz sampling rate during 30 steps on a 20-m walking path (Tsushima et al., 2003).

Mean angular movement changes in a normalized gait cycle were calculated separately for the following regions: trunk (sagittal plane) and the main joints of the lower limbs: ankle-shin (sagittal plane), knee (sagittal plane), and hip (sagittal plane). Gait analysis was done according to the RLA scheme (Rancho Los Amigos Hospital Gait Laboratory System), which is widely used in the analysis of physiological and pathological gait (Perry and Burnfield, 2010). In the first step, the average gait pattern was performed separately in each projection of the studied joint for each participant. Average gait patterns of individual patients with HD used to obtain the averaged gait pattern for the whole study group. The same average gait patterns of individual controls used to obtain the average gait pattern for the whole control group. The peak values of angular movement changes in the normalized walking cycle were analyzed. Normal distribution of variables obtained in the study was assessed using the Pearson's chi-squared test. The significance of differences in mean values of the variables was tested with *t-*tests. Data are presented as mean ± *SD, p* < 0.05 was used to establish statistical significance, and data were analyzed using STATISTICA 10 (StatSoft®) software.

## Results

**Figure 2:**
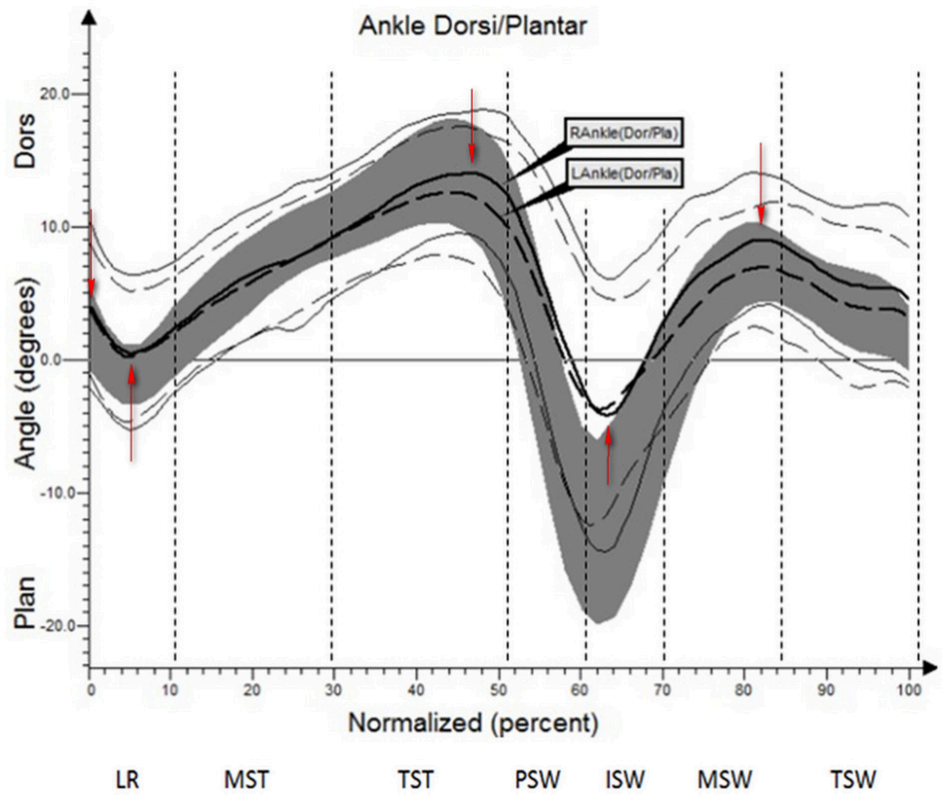
Angular changes in the ankle-shin joint HD study group compared with control group.

**Figure 3:**
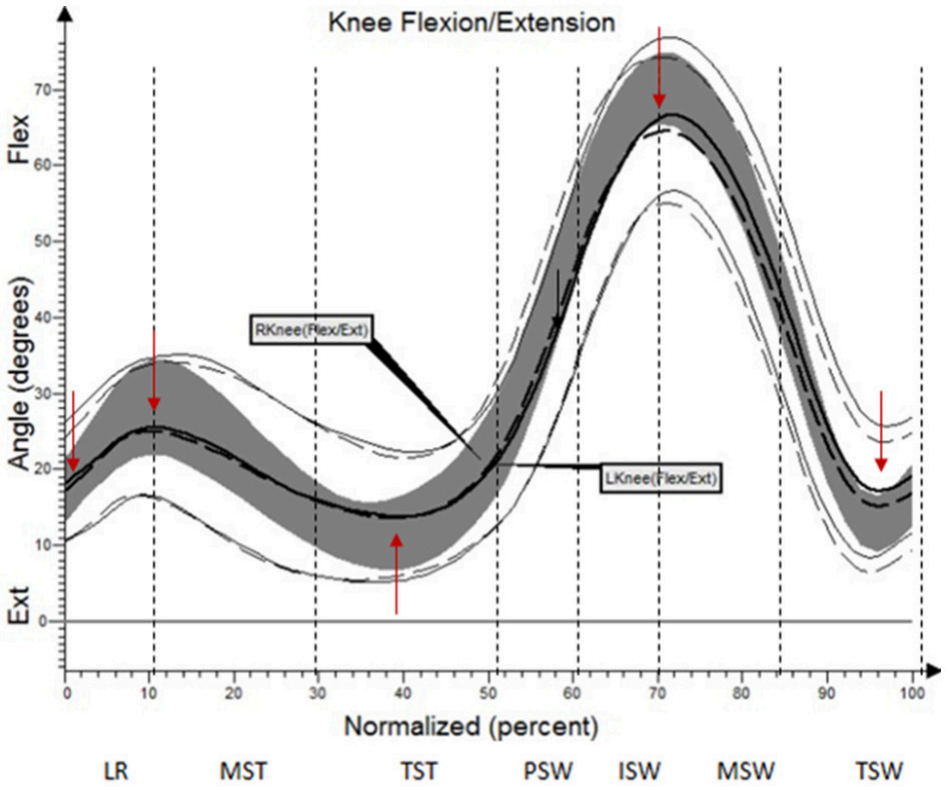
Angular changes in the knee joint HD study group compared with control group.

**Figure 4:**
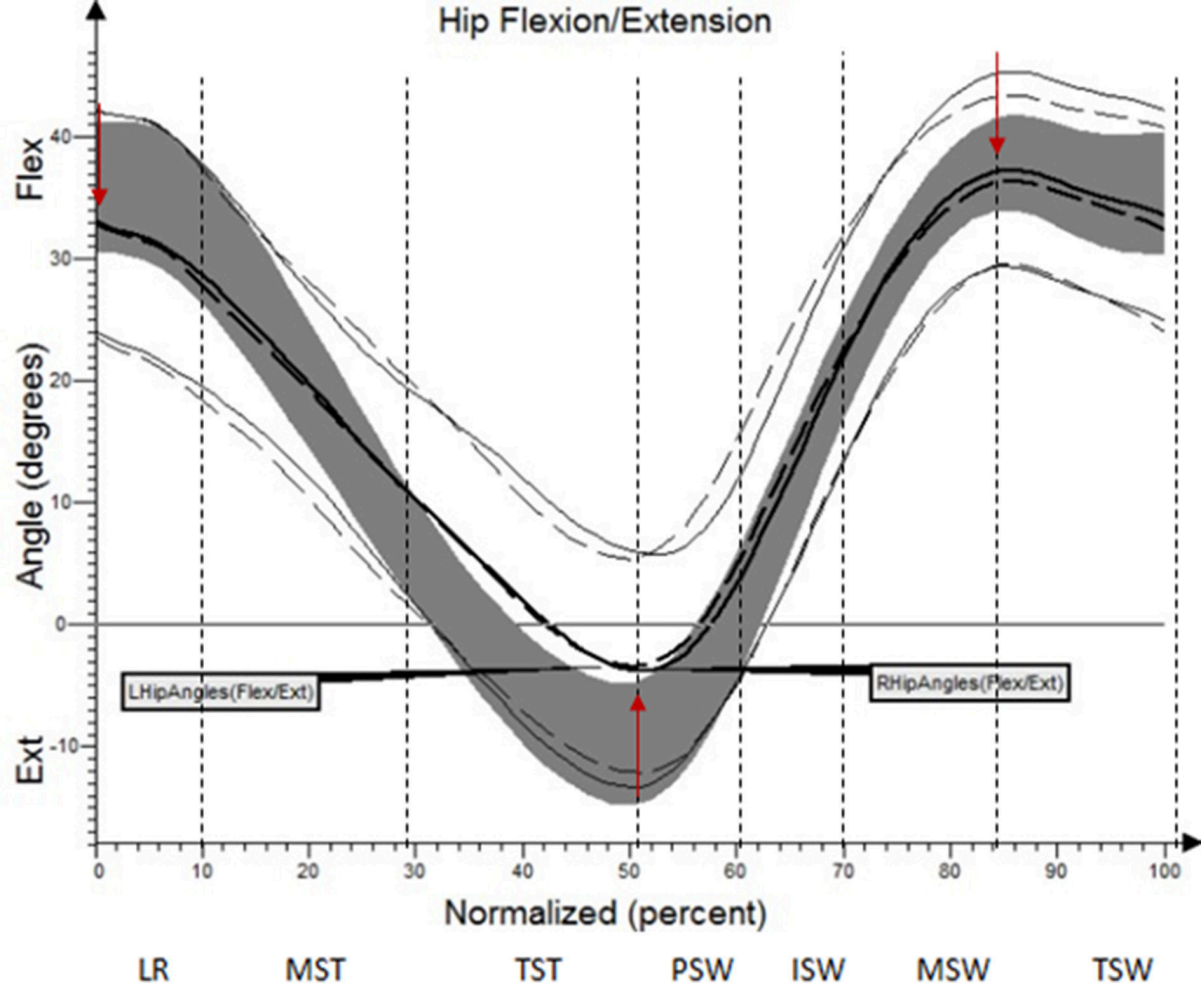
Angular changes in the hip joint HD study group compared with control group.

**Figure 5:**
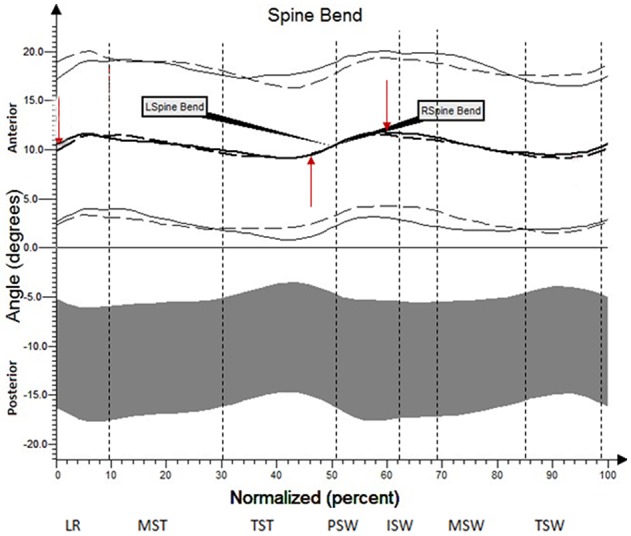
Angular changes in the trunk (spine bend) HD study group compared with control group.

The results (kinematic parameters) are presented on normalized gait cycle charts (histogram), separately for each projection for each joint (Figures 2–**5**). Red arrows indicated in the charts the peak values of angular movement changes subjected to statistical analysis. Vertical dashes lines indicates gait phases: LR (loading response), MST (mid stance), TST (terminal stance), PSW (pre-swing), ISW (initial swing), MSW (mid swing), TSW (terminal swing). Shading area represents double standard deviation of mean angular movement changes of control group. Descriptors (R-right, L-left) of the thick dotted and continuous line refer to mean angular movement changes patients with HD and analogically thin lines refer to standard deviation of this results. The group of subjects with HD achieved higher standard deviation for each measurements compared to the reference group.

The comparison of results regarding the mean angular movement changes of the ankle-shin joint (Figure 2, Table 3) shows statistically significant differences for the left lower limb and the right in phases of Loading Response and Pre-Swing. Significant differences were also found in both knee joints during Initial Swing and Mid Swing phases (Figure 3, Table 4), in both hip joints in Terminal Stance and Pre-Swing phases (Figure 4, Table 5). Significant differences between the groups were found in mean angular movement changes of the trunk in the sagittal plane (spine bend) (Figure 5, Table 6). The values of the spine bend mean angular movement changes were positive in the HD group and negative in the control group throughout the entire analyzed gait cycle.

**Table 3:** Angular changes in the ankle-shin joint compared with control group.

**Limb**	**Gait Phase**	**Angle of movement [°]**				**Statistical significance (*p*)**
		**HD study group**		**Control group**		
		**$\bar{x}$**	***SD***	**$\bar{x}$**	***SD***	
L	IC	3.3	3.3	0.1	3.2	0.0000
L	LR	0.7	4.2	−3.1	3.4	0.0001
L	TST	12.1	8.5	14.4	3.7	0.16
L	ISW	−4.8	6.9	−7.5	5.4	0.04
L	MSW	7.5	4.8	5.5	3.3	0.03
R	IC	4.2	5.2	0.1	3.2	0.0002
R	LR	0.4	5.4	−3.1	3.4	0.001
R	TST	14.6	4.9	14.4	3.7	0.84
R	ISW	−5.1	7.4	−7.5	5.4	0.09
R	MSW	9.5	4.6	5.5	3.3	0.0000

*L, left; R, right, IC, initial contact; LR, loading response; MST, mid stance; TST, terminal stance; PSW, pre-swing; ISW, initial swing; MSW, mid swing; TSW, terminal swing*.

*Red values indicate results statistical significance*.

**Table 4:** Angular changes in the knee joint compared with control group.

**Limb**	**Gait Phase**	**Angle of movement [°]**				**Statistical significance (*p*)**
		**HD study group**		**Control group**		
		**$\bar{x}$**	***± SD***	**$\bar{x}$**	***± SD***	
L	IC	17.7	6.1	16.1	4.2	0.16
L	MST	25.3	7.8	29.8	4.6	0.004
L	TST	12.9	6.6	12.1	4.3	0.48
L	ISW	61.5	17.6	68.9	4.7	0.03
L	TSW	16.9	12.7	11.9	5.3	0.04
R	IC	18.8	6.7	16.1	4.2	0.04
R	MST	26.6	8.1	29.8	4.6	0.04
R	TST	12.7	6.7	12.1	4.3	0.65
R	ISW	65.9	13.5	68.9	4.7	0.26
R	TSW	17.2	9.9	11.9	5.3	0.008

*L, left; R, right; IC, initial contact; LR, loading response; MST, mid stance; TST, terminal stance; PSW, pre-swing; ISW, initial swing; MSW, mid swing; TSW, terminal swing*.

*Red values indicate results statistical significance*.

**Table 5:** Angular changes in the hip joint compared with control group.

**Limb**	**Analyzed parameter**	**Angle of movement [°]**				**Statistical significance (*p*)**
		**HD study group**		**Control group**		
		**$\bar{x}$**	***SD***	**$\bar{x}$**	***SD***	
L	IC	33.2	6.4	38.9	4.2	0.000
L	PSW	−3.0	8.0	−4.3	6.3	0.49
L	TSW	36.4	5.9	39.7	4.6	0.09
R	IC	34.1	5.6	38.9	4.2	0.000
R	PSW	−3.7	7.3	−4.3	6.3	0.69
R	TSW	38.3	5.5	39.7	4.6	0.22

*L, left; R, right; IC, initial contact; LR, loading response; MST, mid stance; TST, terminal stance; PSW, pre-swing; ISW, initial swing; MSW, mid swing; pTSW, terminal swing*.

*Red values indicate results statistical significance*.

**Table 6:** Angular changes in the trunk (spine bend, sagittal plane) compared with controls group.

**Limb**	**Analyzed parameter**	**Angle of movement [°]**				**Statistical significance (*p*)**
		**HD study group**		**Control group**		
		**$\bar{x}$**	***SD***	**$\bar{x}$**	***SD***	
L	IC	10.2	7.4	−10.9	6.3	0.0000
L	TST	7.4	7.4	−11.7	6.2	0.0000
L	PSW	12.9	7.5	−10.6	6.5	0.0000
R	IC	9.5	7.1	−10.9	6.3	0.0000
R	TST	7.2	7.6	−11.7	6.2	0.0000
R	PSW	13.4	7.5	−10.6	6.5	0.0000

*L, left; R, right; IC, initial contact; LR, loading response; MST, mid stance; TST, terminal stance; PSW, pre-swing; ISW, initial swing; MSW, mid swing; TSW, terminal swing*.

*Red values indicate results statistical significance*.

## Discussion

Our research shows a broader view at the problem of gait disturbances in HD paying attention to the setting of the trunk relative to the lower limbs. The results of our study show new aspects of gait analysis in other neurological diseases. The gait pattern in HD is characterized by pathological, over-normative forward inclination of the trunk, resulting in the appearance of compensation patterns in the lower limbs. The upper segment of the body HAT (head, trunk, arms) represents about 62% of the total weight of the body above the pivot axis of the hip joint (Winter and Eng, 2013). Forward inclination of the trunk, in this case, is the greatest drive to walk, which is a direct result of gravity because it creates a component of the force of the gravity moving the whole body. This is of use in clinical practice while physiotherapy may increase stabilization of the trunk in the extension phase of gait (“core-stability”). Core-stability is crucial in maintaining dynamic stability during gait and ensuring safe movement in the intended direction (Tian et al., 1992). The compensations produced in the lower limbs in people with HD are characterized by: insufficient plantar flexion during Loading Response and Pre-swing phases; insufficient flexion of the knee joint during Initial Swing and Mid Swingphases; excessive flexion of the hip in Terminal Stance and Pre-Swing phases. It should be noted that the group of patients with HD obtained higher standard deviation values for all the analyzed mean angular movement changes, which is proof of the high variability within the studied patients.

Thus far, only a few studies dealing with mean angular movement changes in the joints of the lower limbs have been published. The same is true for the analyzed mean angular movement changes of trunk (Tsushima et al., 2003; Fahn et al., 2011). However, no previous groups of respondents were as large as the group in the present study (30 patients with HD). Moreover, the level of significance of the currently available literature is negatively affected by low methodological rigor, small groups of subjects, vague criteria for selection (resulting in potentially heterogeneous groups).

Reynolds et al. analyzed the gait of six persons suffering from HD for whom initial symptoms appeared between the age of 29 and 46. The mean angular movement changes in the ankle-shin, knee, and hip joints were evaluated. The analysis of the results showed that, despite the large variety in gait kinematic profiles of people with HD (only the trajectory of the movement of the ankle was congruous with the reference group), chorea does not impair the realization of gait strategy in any significant way from a purely clinical point of view (Reynolds et al., 1999). The present study observed characteristic problems for the lower limbs in the ankle-shin and knee joints but, from the perspective of the clinical analysis, they are not of key importance in the gait strategy of people with HD.

Delval et al. (2006) were the only authors to have so far conducted simultaneous analysis of kinematic and spatial-temporal parameters of the lower limbs within the same group of HD patients. Delval and his team carried out analysis of the gait disturbances in 15 patients at the early stages of HD as compared to 15 healthy subjects and 15 patients with Parkinson's disease. In patients with HD (as compared to the reference group), they observed a reduction in walking speed and cadence, with a simultaneous lengthening of the cycle time while maintaining correct overall cycle length. The cadence in the group of patients with Parkinson's disease was within norms when compared to the reference group. In addition, an analysis of mean angular movement changes in the lower limb joints of people with HD revealed the concurrence of hypo- and hyper-kinetic disorders. On this basis, the researchers concluded that abnormalities of gait in HD are mainly characterized by disturbances in spatial-temporal parameters, out of which bradykinesia (defined as a decrease in gait speed) is of crucial importance. The results of the present study show that the primary dysfunction is located in the trunk area. In addition, the omission by Delval et al. of any analysis of the “passenger's” behavior in the gait cycle seems to be a significant shortcoming, since correct positioning of the body and its stabilization is essential in maintaining dynamic stability during locomotion in the planned direction (Delval et al., 2006). In the light of the results of the present study and the available literature, postural instability which is very common in HD, could be a potential cause of gait disturbances (Churchyard et al., 2001; Grimbergen et al., 2008). It is probable that problems in planning and sequencing movement can lead to deceleration of locomotion, which in turn is reflected in reduced walking speed (Lawrence et al., 1998; Snowden et al., 2001).

Disturbances of gait pattern in HD are affected by both the neurodegenerative process of the brain and pharmacotherapy itself. Persons qualified for examination took optimized medicine doses in order to obtain the highest level of functionality. Amongst the possible limitations of this original study, there is the obvious fact that pharmacological treatment were neither fully examined nor standardized.

The differences between the results of gait analysis in the present study and in the available literature may result from: different measurement conditions, varying specifications of the gait laboratories, or from the methods used for data collection and processing. For this reason, the results obtained by HD patients are presented against the results obtained by healthy persons. Their gait was evaluated in the same laboratory, using the same Vicon system operated by an experienced staff. Moreover, the differences may also result from the variability of population in different parts of the world. This is confirmed by the reference values for gait patterns in healthy individuals obtained at Jacquelin Perry Musculoskeletal Biomechanics Research Laboratory, CA (Perry and Burnfield, 2010).

Despite the very strong individual characteristics of gait in people with HD, the search for common features of gait disorders aims to create indications for physiotherapy. The stereotypical disorders of gait patterns in Parkinson's Disease have been determined even in cases of very large diversity in gait disorders within the patient (Moreau et al., 2010).

## Conclusion

A characteristic gait disorder common to all patients with HD occurring throughout the whole duration of the gait cycle is a pathological trunk anterior tilt. The results will significantly contribute to programming physiotherapy for people with HD, aimed at stabilizing the trunk in a position of extension during gait. Our research is the first to conduct simultaneous estimation of the mean angular movement changes in the trunk and lower limbs of people with HD, using an objective research tool (three-dimensional motion analysis system-Vicon).

## Author contributions

EM: Study design, data collection, data interpretation, manuscript preparation, and literature search. MF: Data collection, manuscript preparation, and literature search. WC: Statistical analysis, data interpretation. KB: Data collection, medical supervision, and manuscript preparation. MR: Data collection and medical supervision. SP: Manuscript preparation and literature search. JS: Literature search and statistical analysis. AS: medical supervision.

### Conflict of interest statement

The authors declare that the research was conducted in the absence of any commercial or financial relationships that could be construed as a potential conflict of interest.
